# Why nursing cannot be replaced with artificial intelligence

**DOI:** 10.4069/whn.2024.12.12

**Published:** 2024-12-30

**Authors:** Hae-Kyung Jo

**Affiliations:** Department of Nursing, Jeonju University, Jeonju, Korea

## Introduction

In the modern era, generative artificial intelligence (AI), which relies on extensive data sets, is propelling advancements in both culture and technology. AI is acknowledged as an effective system capable of generating content and reducing the costs associated with content creation. As AI becomes integrated into various industries, the boundaries between culture and industry continue to expand [1]. AI is now being actively utilized across multiple sectors including manufacturing, healthcare, transportation, the environment, and education. It is anticipated to enhance national competitiveness and improve the quality of life by increasing convenience. However, ethical concerns related to AI, such as the potential for technology misuse and data bias, have also been raised [2]. AI is gradually being implemented in the medical field, and the deployment of AI and AI nursing robots has been projected to rapidly transform the healthcare landscape [3].

This paper first aims to examine the changes in the healthcare system and nursing care resulting from the introduction of AI. It then considers the current and future roles of nursing in conjunction with AI and ultimately seeks to explore why nursing cannot be fully replaced by AI.

## Changes in the healthcare system and nursing with artificial intelligence applications

AI-based health monitoring devices are currently being used in areas of South Korea (hereafter, Korea) where medical services are underprivileged. Additionally, AI-based medical environments are being developed through the creation of screening programs and disease-prediction telemedicine services [4]. The application of AI in healthcare primarily enhances telemedicine, encompassing services such as remote patient monitoring, consultations, surgeries, and rehabilitation. The coronavirus disease-2019 pandemic has underscored the benefits of a contactless social life and healthcare system, highlighting their convenience and rationality [5]. In Korea, the Telemedicine Industry Council conducted a survey among 1,506 patients, 113 doctors, and 161 pharmacists who had experienced non-face-to-face treatments during the first year of a pilot project. The results showed that 93.2% of patients were satisfied and reported utilizing the system when access to medical care was weak [6]. AI is also widely used with primary care health records, connecting data with other data to enable active care. While the use of AI is currently limited to data science or electronic medical records in medical and nursing education curricula, it provides a decision support system at scale that will increase learning capacity and transform the future of healthcare [7]. AI is already being implemented for disease diagnosis and prognosis, treatment optimization and outcome prediction, drug development, and public health [4]. It plays a crucial role in preventing burnout among nurse practitioners by aiding in scientific decision-making. Given patients’ preferences for non-face-to-face care, along with the potential to strengthen community health, streamline the workload of doctors and nurses, and improve scientific decision-making in medical and nursing practices, the integration of AI in healthcare is anticipated to grow rapidly. However, the advancement of AI technology necessitates the collection and sharing of vast amounts of data, raising concerns about personal information protection. It is also crucial to address potential damages and ethical issues associated with AI applications [2].

## The present and future prospects of nursing with artificial intelligence

AI nursing technology is predicted to have a positive impact on the medical field in the future. In fact, the Organization for Economic Co-operation and Development’s (OECD) AI Policy Observatory is attracting more investment in the AI healthcare industry than in any other sector [8]. The Korean government is implementing an AI support project to assist public medical institutions in introducing and utilizing medical healthcare solutions and services that involve digital technology [2]. Nursing services are expected to expand beyond medical institutions to include communities, homes, and facilities through patient-centered medical services. Therefore, the scope of nurses’ roles is anticipated to increase dramatically compared to the past [9]. Nursing care is already transitioning from being centered around medical institutions to focusing on community-based services. As a result, the role of nurses, as well as the proportion and scope of nursing work, will significantly increase. However, there has recently been a growing imbalance between the supply and demand of nurses in Korea [10]. For new nurses, this often results in difficulty adjusting to the workplace, with many facing excessive workloads and complex interpersonal relationships. There is a pressing need to support both new and existing nurses to adapt effectively to the clinical environment and develop into skilled nursing professionals. Consequently, it is legally mandated to have a designated nurse in charge of education to enhance nurses’ capabilities and improve the quality of medical care [11]. This initiative also reflects the Korean government’s commitment to supporting the expansion of nurses’ duties and roles.

Collaboration between medical and nursing professionals with AI is already underway. AI-driven medical voice services are alleviating the burden on medical staff and nurses by streamlining patient information services during hospital visits. This integration extends to linking electronic medical records and electronic nursing records with the system, thereby reducing the workload associated with documentation for nurses [12]. In the field of nursing, AI is utilized for nursing diagnoses. AI prediction models identify patients at high risk of falling, and a clinical decision-supporting system is employed to categorize the severity of emergency patients. Additionally, a severity classification score, known as the Korea Triage and Acuity Scale, is implemented during the nurse’s initial assessment [13]. These changes in the adoption of AI in nursing are heralding a variety of growth in the work of nurses.

The future of nursing with AI is bright. AI will not only reduce medical costs but also enhance the level of medical support. This advancement will facilitate the early diagnosis of diseases, enable the investigation and prediction of clinical trends, manage and optimize medical resources, and ultimately improve the quality of care. For instance, AI can improve communication between nurses and patients, thereby reducing nurse fatigue and burnout. It will minimize resource waste by cutting down on paper use, decrease patient waiting times, and enhance the quality of health education and nursing care through better appointment management and scheduling [14]. In terms of optimizing teaching, simulation-based practical training for nursing students and hospital education can be effectively implemented [4]. AI will also improve nursing education and research, increasing the knowledge base of nurses and undergraduates regarding nursing practices. Additionally, AI can evaluate perfusion in ulcer and pressure ulcer wound healing [15]. Continuous monitoring of pregnant women with diabetes will benefit both maternal and fetal health, potentially improving outcomes in women’s health [14]. AI can also protect patients by reducing the risk of falls and assisting in the care of patients with sleep apnea [16]. It will provide early warnings of patient deterioration, thereby improving recovery rates [17]. The use of AI technology will enable the documentation of nurses’ experiences in case form, lead to the development of new nursing diagnoses, and allow for the collection of more data through the study and analysis of resolved cases. The benefits outlined above will significantly advance the future of nursing and accelerate the adoption of AI in the field.

## Why artificial intelligence cannot replace nursing

The future of nursing with AI promises to improve the quality of care by reducing costs, improving medical treatment, aiding research through the prediction of clinical trends, optimizing resource management, and preventing nurse burnout [14]. Despite these numerous benefits and promising prospects, ethical considerations are crucial in determining why AI cannot replace human nursing. To effectively integrate AI into nursing, it is essential to understand and adhere to AI’s ethical standards [2]. First, regarding the principle of human dignity, AI must be developed and utilized in ways that preserve human life and safeguard mental and physical health. It should be safe and robust, ensuring no harm comes to humans. Consequently, a patient receiving care from an AI robot or machine must give their mental consent to the AI-provided care. Patients need to feel comfortable with AI care, experience no physical discomfort, and trust that their health will not be compromised. Currently, there is no established process for patients to express their willingness to accept AI care or to communicate their emotional and physical responses during its provision. Second, the principle of the common good requires that AI be accessible to socially disadvantaged and vulnerable groups, who are at risk of being marginalized in the intelligent information society. It is crucial to assess whether AI assists these vulnerable groups and whether AI-provided nursing care is suitable. When providing information, monitoring participant responses, posing questions, re-learning, and offering nursing care that involves sensitive and empathetic dialogue with vulnerable clients are necessary. The more socially underprivileged and vulnerable individuals are, the less likely they are to access AI due to financial constraints and limited opportunities for AI-related education. AI tends to benefit those with higher educational and economic statuses, making AI nursing more challenging for the socially underprivileged and vulnerable groups compared to direct nursing. Third, the issue extends beyond ethics to delegation. According to the American Nurses Association (ANA), nursing delegation involves transferring the responsibility for performing a task from one individual to another while retaining accountability for the outcome [18]. AI robots cannot be considered ‘individuals’ as they lack personhood. Moreover, according to the ANA’s five rights of nursing, the first right is “the right task,” which involves matching the task with the right person based on their scope of practice, competencies, and skill levels [19]. When delegating, the task should be assigned to a person evaluated by the nurse for the necessary qualifications and experience, reflecting the “right situation” and “right person,” the second and third rights, respectively. The “right situation” involves considering timing and urgency to minimize risks, where AI can be effective, but full delegation is not feasible. The “right person” requires that the delegating nurse has confidence in the competence of the delegated nurse, who must possess the requisite knowledge, skills, and education. Delegation also necessitates trust within the healthcare team and the ability to communicate openly. However, AI lacks the capability for open communication, including contextual and nonverbal interactions.

Fourth, this issue relates to the nature of nursing. Fundamentally, nursing revolves around the concept of care, which necessitates compassion and sensitivity. This care process involves mutual communication, emphasizing the importance of human interaction. However, AI lacks the consciousness required for subjective experience [11] and does not possess the emotional or ethical framework needed for moral judgment or a sense of responsibility. The concept of human dignity in the nurse-patient relationship during the provision of care is inherently human. While AI can be trained to recognize certain human emotions, it cannot predict or respond appropriately to a patient’s emotional and physical states in varying situations. At its core, caring is the essential, central, and integrative act that defines nursing. It involves assisting, supporting, or facilitating individuals or groups who have a present or potential need to improve or enhance their condition, lifestyle, or way of life [20]. Thus, nursing care, which addresses the physical, mental, spiritual, and social well-being of individuals, involves making scientific decisions based on a deep understanding of the patient’s subtle expressions, words that may not match their emotions, and even their hidden intentions.

## Conclusion

No matter how advanced and intelligent AI becomes, it cannot replace the core aspect of nursing, which is the care that necessitates human emotions and judgments. AI lacks the capacity to embrace the nursing philosophy that is grounded in human dignity and cannot be held accountable for its actions. Moreover, AI is limited in its ability to make ethical decisions or to quickly assess and respond to emerging situations in clinical settings, making true delegation impossible.

In conclusion, while AI may serve a supportive role, it cannot replace the role of nursing. The integration of AI into nursing necessitates a profound consideration of human dignity and an understanding of AI ethical standards. Moreover, the principle of the common good must be maintained, particularly for socially disadvantaged and vulnerable groups. It is important to remember that delegation refers to the transfer of nursing tasks to non-AI personnel, along with the associated responsibilities. Additionally, there must be a continuous dialogue about the nature of AI judgments that could potentially compromise the essence of nursing and human dignity.
